# Biomarkers of oxidative stress and its nexus with haemoglobin variants and adverse foeto-maternal outcome among women with preeclampsia in a Ghanaian population: A multi-centre prospective study

**DOI:** 10.1371/journal.pone.0283638

**Published:** 2023-03-30

**Authors:** Ganiwu Abdul, William Osei-Wusu, Gordon Akuffo Asare, Samira Daud, Stephen Opoku, Valentine Christian Kodzo Tsatsu Tamakloe, Joseph Frimpong, Benedict Sackey, Wina Ivy Ofori Boadu, Vivian Paintsil, Max Efui Annani-Akollor, Yaw Amo Wiafe, Enoch Odame Anto, Otchere Addai-Mensah

**Affiliations:** 1 Department of Medical Diagnostics, Faculty of Allied Health Sciences, Kwame Nkrumah University of Science and Technology, Kumasi, Ghana; 2 University Clinic, University Health Directorate, University of Energy and Natural Resources, Sunyani, Ghana; 3 Department of Medical Diagnostics, College of Health and Wellbeing, Kintampo, Ghana; 4 Department of Haematology, Faculty of Allied Health Sciences, University for Development Studies, Tamale, Ghana; 5 Department of Molecular Medicine, School of Medicine and Dentistry, Kwame Nkrumah University of Science and Technology, Kumasi, Ghana; 6 Department of Child Health, Komfo Anokye Teaching Hospital, Kumasi, Ghana; 7 Centre for Precision Health, School of Medical and Health Sciences, Edith Cowan University, Joondalup, Western Australia, Australia; University of the Witwatersrand, SOUTH AFRICA

## Abstract

**Introduction:**

Haemoglobin variants and preeclampsia (PE) are associated with adverse fatal events of which oxidative stress may be an underlying factor. Oxidative stress (OS) among preeclamptic women with haemoglobin variants has been well established. It is, however, unclear whether haemoglobin variants induce OS to aggravate the risk of adverse foeto-maternal outcomes in pregnant women with preeclampsia. We measured the levels of OS biomarkers and determined the association between haemoglobin variants, and adverse foeto-maternal outcomes among pregnant women with PE.

**Methods:**

This multi-centre prospective study recruited 150 PE women from three major health facilities in both Bono and Bono east regions of Ghana from April to December 2019. Haemoglobin variants; HbAS, HbSS, HbSC, HbCC, and HbAC were determined by haemoglobin electrophoresis. OS biomarkers such as malondialdehyde (MDA), catalase (CAT), vitamin C, and uric acid (UA) along with haematological and biochemical parameters were estimated using standard protocol. Adverse pregnancy complications (APCs) such as post-partum haemorrhage (PPH), HELLP (Haemolysis, Elevated liver enzymes, Low platelet count) syndrome, preterm delivery, neonatal intensive care unit (NICU) admission, and neonatal jaundice were recorded.

**Results:**

Of the 150 pregnant women with preeclampsia, the distribution of haemoglobin AA, AS, AC, CC, SS and SC phenotypes were 66.0%, 13.3%, 12.7%, 3.3%, 3.3% and 1.3%, respectively. The most prevalent foeto-maternal outcomes among PE women were NICU admission (32.0%) followed by PPH (24.0%), preterm delivery (21.3%), HELLP syndrome (18.7%), and neonatal jaundice (18.0%). Except for vitamin C level which was significantly higher in patients with at least a copy of Haemoglobin S variant than those with at least a copy of Haemoglobin C variant (5.52 vs 4.55; *p* = 0.014), levels of MDA, CAT, and UA were not statistically significantly different across the various haemoglobin variants. Multivariate logistic regression model showed that participants with HbAS, HbAC, having at least a copy of S or C and participants with HbCC, SC, SS had significantly higher odds of neonatal jaundice, NICU admission, PPH and HELLP syndrome compared to participants with HbAA.

**Conclusion:**

Reduced levels of vitamin C are common among preeclamptics with at least one copy of the HbC variant. Haemoglobin variants in preeclampsia contribute to adverse foeto-maternal outcomes with Haemoglobin S variants being the most influencing factor for PPH, HELLP, preterm labour, NICU admission, and neonatal jaundice.

## Introduction

Haemoglobinopathies are red cell genetic abnormalities that are widespread in various parts of the world especially Africa, the Mediterranean region, and Asia [1]. The most common and clinically important haemoglobinopathies are due to inadequate production of beta chains; beta-thalassemia or the production of abnormal beta chains (Hb variant) [2]. The commonest and medically important Hb variants include HbS and HbC [3, 4] with geographical distribution of about 1–10% HbSS, 15–30.5% HbAS and < 1% SC in Africa. It has been estimated that 2% of Ghanaian newborns have sickle cell disease with a reported significant number of pregnant women having the disease [5].

Pregnant women are predisposed to immune factors such as oxidative stress which may cause placental dysfunction. The placental dysfunction in turn causes the placenta to release soluble anti-angiogenic factors such as soluble FMS-like tyrosine kinase-1 (sFLT-1), soluble endoglin (sEng), and other inflammatory mediators into the maternal circulation, which induces global endothelial dysfunction leading to preeclampsia.

Preeclampsia (PE) is a pregnancy-related complication characterized by the inception of hypertension with proteinuria or multiorgan impairment at or after week 20 of pregnancy [6]. Although PE has an unknown cause, its pathophysiology is believed to occur through syncytiotrophoblast damage and local placental hypoxia, which leads to increased oxidative stress (OS) [7, 8] and subsequent adverse maternal, fetal, and neonatal complications [9]. Pregnant women with Sickle cell disease (SCD) are associated with an increased risk of stillbirth, small for gestational age, preterm delivery, and maternal mortality compared to the normotensive women [10]. In addition, pregnant women with SCD had been reported to have a higher risk of developing pregnancy-related complications such as venous thromboembolism, intrauterine fetal demise, and intrauterine growth restriction [11]. Moreover, increased levels of HbF are found in the placenta of women who have preeclampsia associated with fetal growth restriction (IUGR) [12]. Increased fetal hemoglobin by the placenta leading to increased consumption of endogenous heme scavenging proteins which implicates the pathogenesis of preeclampsia co-existing with IUGR and the risk of future cardiovascular diseases and chronic conditions [12].

However, in Africa, however, the extent to which haemoglobin variants and immune factors such as oxidative stress influence the adverse foeto-maternal outcome is lacking. We, therefore, evaluated the influence of haemoglobin variants and oxidative stress on adverse foeto-maternal outcome in preeclamptic women in the Bono Regions of Ghana.

## Materials and methods

### Study design and sites

This multi-centre prospective study involving three healthcare facilities, namely, Techiman Holy Family hospital, Berekum Holy Family hospital, and Bono Regional Hospital was conducted from April to December 2019. Reports from the Regional Health Directorates indicate that these hospitals together consistently record about 70% ANC attendance in the entire Region. Again, these hospitals have obstetrician/gynaecologists and pediatricians and so receive referral from the Northern, Western and Ashanti regions.

### Study participants and recruitment

A purposive sampling technique was used to recruit a total of 150 singleton pregnant women aged between 17–45 years who were clinically diagnosed of preeclampsia by specialist/consultant obstetric and gyaenocologists. Information of sociodemographic, clinical history, obstetrics information and body mass index (BMI) of the participants were obtained from the patients’ folders. Preeclampsia was defined based on the revised definition by International Society for the study of Hypertension in pregnancy (ISSHP) as new-onset of gestational hypertension (≥140 mmHg systolic/≥90 mmHg diastolic) developed at or after 20 weeks’ gestation and with new-onset of at least one of proteinuria maternal organ dysfunctions (neurological complications, pulmonary oedema, haematological complications, liver involvement or acute kidney injury) and or uteroplacental dysfunction [13].

Diagnosis of preeclampsia was done by consultant obstetrician and gynaecologist. Uteroplacental dysfunction was evaluated with ultrasound assessment of foetal growth and umbilical artery Doppler velocimetry or cerebroplacental ratio measurements to assess blood flow redistribution in placental insufficiency. A total of 150 participants comprising 87, 33, 30 preeclamptic women were recruited from Techiman Holy Family, Bono Regional, and Berekum Holy Family Hospitals, respectively. These proportions were based on the proportion of antenatal care attendance from April to December 2019. Preeclamptic women including nulliparous, primiparous and multiparous in their second and third trimesters of pregnancy were recruited for the study. Pregnant women without preeclampsia those with already known renal diseases, diabetes mellitus, gestational hypertension, chronic hypertension, and twin pregnancies were excluded from the study.

### Sample size justification

The sample size for this study was determined based on the prevalence of preeclampsia 10% among pregnant women at the Bono Regional Hospital (Brong Ahafo Regional Hospital annual report, 2010). At a 95% confidence level and 5% margin of error, a sample size of 138 was obtained using the Cochrane’s formula; n = z^2^pq / d^2^. A minimum of 150 were finally enrolled comprising of 87, 33, 30 preeclamptic women from the Techiman Holy Family, Bono Regional, and Berekum Holy Family Hospitals, respectively. These proportions were based on the total proportion of antenatal care attendance. The annual antenatal care attendance was 30,467 for Techiman Holy Family Hospital 11,557 for Brong Ahafo Regional Hospital and 10,506 for Berekum Holy Family Hospital.

### Sample collection and processing

Urine samples (10–20 ml of urine) were collected into sterile, leakproof urine containers and proteinuria was measured using dipstick protein analysis (URIT 10V urine reagents, Medical Electronic Co., Ltd China). BD Vacutainer syringe and needle was used to collect venous blood (10 milliliters (mls)) venous blood of which 5 mls was dispensed into labelled vacutainer^®^ plain tubes and 5 mls into Ethylene Diamine Tetra-acetic Acid (EDTA) vacutainer blood collection tube. Samples collected into the EDTA tube were used for full blood count, sickling testing, and haemoglobin electrophoresis. Haematological profile of study participants was estimated using a five (5)-parts fully automated haematology analyser (URIT-5250, China) whiles haemoglobin variants were estimated using sickling test and electrophoresis by cellulose acetate membrane (CAM).

Samples collected into the vacutainer^®^ plain tubes were then centrifuged at 4000 rpm for 10 minutes. The generated sera obtained from the span blood were immediately analysed or where there was delay in testing samples were collected into a labelled fresh sterile, acid-washed, plain capped bottles and stored at −20°C and analysed within 24 hours. The generated sera obtained from the span blood were stored at −20°C until analysis. Serum malondialdehyde (MDA), catalase (CAT), and vitamin C were assayed using spectrophotometer (Biomate 3S UV Visible spectrophotometer, Thermoelectron Inc USA) and uric acid (UA) was assessed using fully automated chemistry analyser (DIASYS Respons^®^ 920 Chemistry Analyzer).

### Oxidative stress biomarker assay

MDA levels were measured by the MDA thiobarbituric acid (TBA) test by means of colorimetric reaction of MDA and TBA in acid solution. MDA, as secondary product of lipid peroxidation, reacts with TBA to produce a red-coloured product, which was detected spectrophotometrically at 535 nm. The protocol used in our study was described by Kamal *et al*. [14], which is a modified version of the Shlafer and Shepard protocol [15]. The absorbance of the mixture was measured at 535 nm with a spectrophotometer (Biomate 3S UV Visible spectrophotometer, Thermoelectron Inc USA) and the results expressed as μmol/l, using the extinction coefficient of 1.56×105 L mmol-1 cm1. The concentration of MDA (nmol/ml) was calculated by using the following formula: Concentration of the test = Abs (test)–Abs (blank) / 1.56 x 1000000.

Catalase was measured by the method described by Takahara *et al*, [16]. Plasma (0.2 ml) was added to 1.2 ml of 50 mM phosphate buffer (pH 7.0). The enzymatic reaction was initiated by the addition of 1.0 ml of 30 mM H2O2 solution. The decrease in absorbance was assayed at 240 nm at 30 seconds time intervals for 3 minutes with an ultraviolet visible spectrophotometer (Biomate 3S UV Visible spectrophotometer, Thermoelectron Inc USA). Enzyme blank was run simultaneously with 1.0 ml of distilled water instead of hydrogen peroxide. The enzyme activity was expressed as units/ml.

Vitamins C is described as an antioxidant against oxidative stress and therefore have reduced antioxidant capacity [17, 18]. Vitamin C was determined by the method of Omaye *et al*., [19] at most 3 hours after sample collection. Ascorbic acid in plasma is oxidized by Cu (II) to form dehydroascorbic acid, which in turn reacts with acidic 2, 4- dinitrophenylhydrazine to form a reddish dihydrazone which was then measured at 520 nm with a spectrophotometer (Biomate 3S UV Visible spectrophotometer, Thermoelectron Inc USA).

### Postpartum assessment of maternal and perinatal outcomes

All maternal and perinatal outcomes were recorded for participants after baby delivery. Obstetric outcomes that were assessed included gestational age at delivery, mode of delivery, preterm delivery (defined as a delivery < 37 weeks), premature rupture of membrane (PROM), antepartum haemorrhage (APH), Postpartum haemorrhage (PPH), intrauterine growth restriction (IUGR), placenta previa, intrauterine fetal death (IUFD), intrapartum stillbirth and Haemolysis Elevated Liver Enzymes and Low Platelet (HELLP) syndrome. Perinatal outcomes assessed included gestational age at delivery, birth weight at delivery (low birth weight [LBW] defined as birth weight < 2.5 kg), Apgar score, neonatal weight, Neonatal Intensive Care Unit (NICU) admission, complications such as neonatal jaundice, and neonatal death (mortality).

### Ethical consideration

Approval for this study was obtained from the Committee on Human Research, Publications and Ethics of the School of Medicine and Dentistry, KNUST (CHRPE/AP/693/19) and the Ethical review committee of the three Hospitals. Participants gave written informed consent before the commencement of the study.

### Statistical analysis

Data was entered into Microsoft Excel 2010 and analysed using the SPSS software (IBM SPSS Statistics version 20; Chicago, IL, USA) and Windows SAS software (SAS for Windows version 9.4, SAS Institute Inc, Cary, NC). The Kolmogorov-Smirnov test was used to test the normality of the data distribution. Data was presented as frequency (proportion) for categorical variables and mean ± SD for continuous parametric variables. One-Way ANOVA followed by Tukey Post-hoc multiple comparison test was performed to compare between haematological and oxidative stress markers across the haemoglobin variants. The SPSS software was used to perform the One-Way ANOVA analysis. The univariate and multivariate binary logistic regression was used to assess the association between haemoglobin variants and foeto-maternal outcomes. The univariate binary logistic regression analysis models were used to assess association between maternal outcomes such as the development or otherwise of PPH (“Developed” versus “Not developed”), HELLP Syndrome (“Developed” versus “Not developed”) and Pre-term birth (“Yes” versus “No”); against Haemoglobin variants such as HbAA, HbAS, HbAC, at least a copy of HbS and at least a copy of HbC. The multivariate logistic regression was performed using the stepwise forward selection criteria. The multivariate logistic regression was adjusted for maternal age, number of births and residence. The SAS software for windows was used to perform logistic regression analysis. P-values ˂ 0.05 were considered statistically significant.

## Results

### Descriptive summary of socio-demographic data of the study participants

The demographic characteristics of the study participants are presented in Table 1. Almost one-half of the study participants were 26–30 years (40.0%), had had secondary education (40.0%), or were from urban areas (46.0%). More than half were married (60.7%), were living in rural areas (54%) or were Christians (74.7%). In terms of occupation status, more than one-third were traders (36.7%) whilst farmers were the least (10.0%). Most study participants at the time of presentation were carrying their second pregnancy (40.0%) whilst (20.0%) were carrying pregnancy for the first time.

**Table 1:** Socio-demographic characteristics of the study participants.

Variable	Category	Frequency (n = 150)	Percentage (%)
**Age (years)**	18–25	27	18.0
	26–30	60	40.0
	31–35	32	21.3
	>35	31	20.7
**Marital status**	Married	91	60.7
	Single	50	33.3
	Other	9	6.0
**Educational level**	No formal education	8	5.3
	Basic	36	24.0
	Secondary	60	40.0
	Tertiary	46	30.7
**Residence**	Urban	69	46.0
	Rural	81	54.0
**Religion**	Christians	112	74.7
	Muslim	32	21.3
	Traditionalist	6	4.0
**Occupation**	Farmer	15	10.0
	Public service	41	27.3
	Trader	55	36.7
	Unemployed	39	26.0
**Gravidae**	1^st^	30	20.0
	2^nd^	60	40.0
	3^rd^	42	28.0
	4^th^ or more	18	12.0

### Distribution of haemoglobin variants among study participants

**Fig 1:**
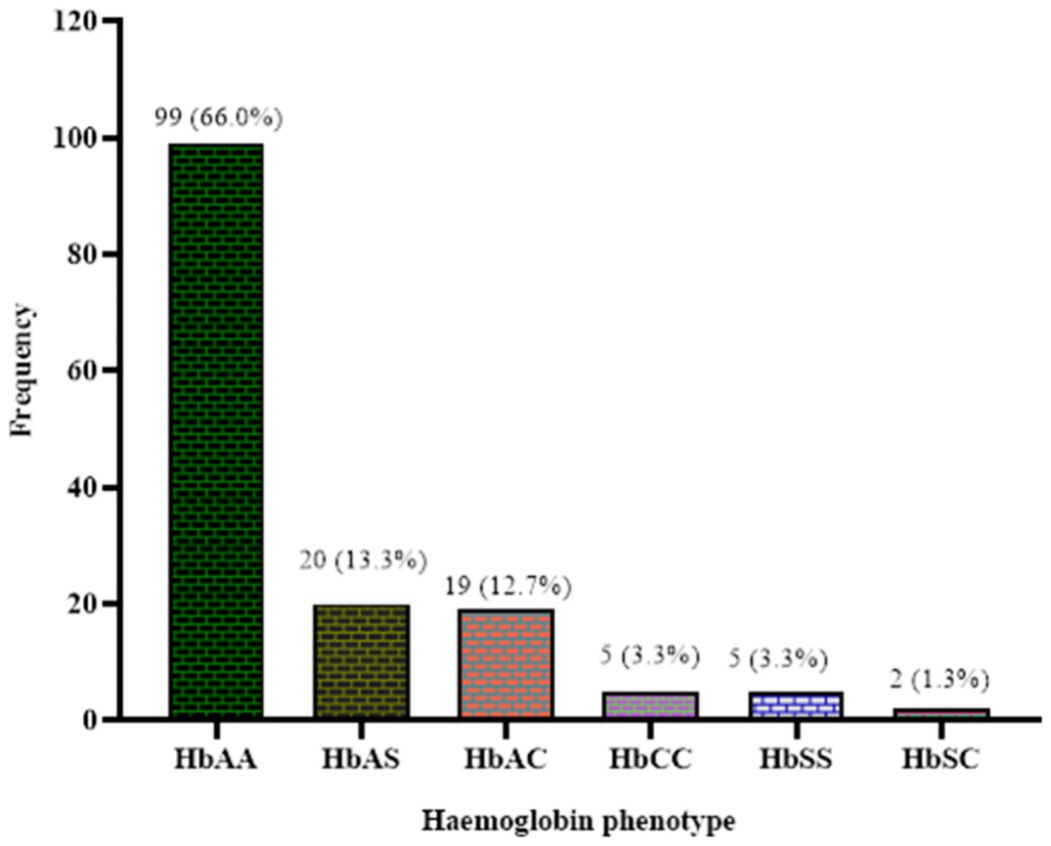
Distribution of haemoglobin phenotypes among the study participants.

The distribution of haemoglobin variants among the study participants is shown in Fig 1. Majority of the study participants had HbAA (66.0%). The proportion of individuals with HbAS and HbAC were (13.3%) and (12.7%) respectively. Autosomal recessive phenotypes (HbSS and HbCC) were (3.3%) for each of the study participants. A few of the study participants had HbSC phenotype (1.3%) (Fig 1).

### Difference in the levels of haematological and oxidative stress markers across the haemoglobin variants

The distribution of haematological and oxidative stress markers with haemoglobin variants are shown in Table 2. The mean WBC was significantly higher among participants with other haemoglobin variants (Hb SS, SC, CC) compared to either HbAA, HbAS or HbAC (*p* = 0.007). The mean haematocrit was significantly higher among participants with HbAA phenotype compared with those with HbAS, HbAC and the other variants (HbSS, SC, and CC) (*p* < 0.0001). Also, the mean vitamin C levels were higher among HbAS individuals compared with HbAA and HbAC phenotype (*p* = 0.001). Except for WBC, hematocrit and vitamin C which were significantly different across the haemoglobin variants, there was no significant difference in the other haematological, oxidative stress and BMI variables (p>0.05).

**Table 2:** Distribution of haematological and oxidative stress markers across the haemoglobin variants.

Variable	Haemoglobin Variant				*p*-value
	HbAA (n = 99)	HbAS (n = 20)	HbAC (n = 19)	Others (n = 12)	
**Gestational BMI**	22.15±2.59	22.29±3.55	21.95±3.31	22.55±3.25	0.950
**CBC**					
HGB (g/dl)	12.78±1.19	13.02±1.36	13.43±0.79	13.44±0.82	0.050
WBC (10^3/Ul)	7.36±2.20^a^	6.77±1.80^a^	7.26±2.33^a^	10.10±6.58^b^	**0.007**
RBC (10^6^/μl)	3.81±0.48	3.91±0.63	3.97±0.43	4.07±0.37	0.218
PLT (10^3/uL)	231.23±66.09	219.0±77.61	255.47±66.19	213.50±64.13	0.274
HCT %	38.38±4.45^b^	34.75±3.60^a^	34.45±5.02^a^	33.99±5.43^a^	**<0.0001**
MCV (fL)	97.30±7.32	96.01±5.08	96.11±5.93	96.12±7.55	0.784
MCH (pg)	34.45±3.14	34.06±4.06	33.09±2.89	32.80±3.51	0.192
MCHC (g/dl)	35.49±3.40	35.46±3.57	34.82±3.41	34.0±1.99	0.461
RDW	11.70±2.31	12.19±1.34	11.83±1.52	11.68±0.76	0.806
RDW-SD (fL)	73.60±10.47	70.97±10.98	73.14±8.22	69.69±8.82	0.497
RDW-CV %	12.05±5.71	12.19±1.34	11.83±1.52	11.68±0.76	0.989
**Oxidative markers**					
MDA (nmol/ml)	4.35±0.98	4.62±0.91	4.25±0.94	4.79±1.09	0.307
Catalase (U/mL)	5.00±0.66	5.16±0.85	5.10±0.83	5.26±0.66	0.548
Vitamin C (mg/ml)	4.91±1.17^a^	5.78±1.12^b^	4.29±0.94^a^	4.90±1.22^a, b^	**0.001**
Uric Acid (mg/dL)	7.53±0.57	7.26±0.52	7.51±0.42	7.40±0.43	0.194

MDA (Malondialdehyde), HB(Haemoglobin), MCHC (mean corpuscular haemoglobin concentration), MCH (mean corpuscular haemoglobin), RBC (red blood cell count), HCT (haematocrit), MCH (mean cell haemoglobin), WBC (white cell count), MCV (mean cell volume), PLT (platelets), Neutrophils, Lymphocytes, MON (Monocytes), EOS (Eosinophils), BASO (Basophils), Red blood cell distribution width, coefficient of variation (RDW-CV), red cell distribution width—standard deviation (RDW-SD) and Body mass index (BMI)

### Distribution of haematological and oxidative stress markers across the three dominant haemoglobin variants

Table 3 shows the distribution of haematological and oxidative stress markers across the three dominant haemoglobin variants. Compared with HbAA phenotype, individuals with at least one copy of HbC variant had significantly higher Hb levels (13.41 vs 12.78, *p* = 0.036). The mean haematocrit level was significantly higher (*p* <0.0001) in HbAA individuals than those having at least a copy of the recessive variants (either HbS or HbC). Moreover, the mean vitamin C levels were significantly higher in patients with at least a copy of HbS variant than those with at least a copy of HbC variant (5.52 vs 4.55, *p* = 0.014). Except for haemoglobin, hematocrit and vitamin C which were significantly different across the three dominant haemoglobin variants, there was no significant difference in the BMI and the other haematological, and oxidative stress variables (p>0.05).

**Table 3:** Distribution of haematological and oxidative stress markers across the three dominant haemoglobin variants.

Variables	Haemoglobin Variants			*p*-value
	HbAA (N = 99)	At least one copy of HbS variant	At least one copy of HbC variant	
**BMI**	22.15±2.59	22.44±3.39	22.26±3.36	0.903
**CBC**				
HGB(g/dl)	12.78±1.19^a^	13.04±1.24^a,b^	13.41±0.80^b^	**0.036**
WBC (10^3/Ul)	7.36±2.20^a^	8.00±4.95	7.59±2.38	0.604
RBC (10^6^/μl)	3.81±0.48	3.98±0.60	3.95±0.41	0.190
PLT (10^3/uL)	231.23±66.09	212.04±71.89	252.83±69.60	0.111
HCT %	38.38±4.45^b^	33.76±4.34^a^	35.08±4.89^a^	**<0.0001**
MCV (fL)	97.30±7.32	95.67±6.21	96.65±5.91	0.563
MCH (pg)	34.45±3.14	33.62±4.21	33.37±2.73	0.250
MCHC (g/dl)	35.49±3.40	35.12±3.24	34.77±2.26	0.610
RDW	11.70±2.31	12.14±1.24	11.77±1.39	0.631
RDW-SD (fL)	73.60±10.47	71.58±9.95	71.77±9.35	0.559
RDW-CV %	12.05±5.71	12.14±1.24	11.77±1.39	0.958
**Oxidative stress markers**				
MDA (nmol/ml)	4.35±0.98	4.68±1.02	4.37±0.95	0.326
Catalase (U/mL)	5.00±0.66	5.21±0.83	5.14±0.77	0.347
Vitamin C (mg/ml)	4.91±1.17^a,b^	5.52±1.24^b^	4.55±1.10^a^	**0.014**
Uric Acid (mg/dL)	7.53±0.57	7.30±0.52	7.48±0.54	0.161

MDA (Malondialdehyde), HB(Haemoglobin), MCHC (mean corpuscular haemoglobin concentration)-MCH (mean corpuscular haemoglobin), RBC(red blood cell count), HCT (haematocrit), MCH (mean cell haemoglobin), WBC (white cell count), MCV (mean cell volume), PLT (platelets), Neutrophils, Lymphocytes, MON (Monocytes), EOS (Eosinophils), BASO (Basophils), Red blood cell distribution width, coefficient of variation (RDW-CV), red cell distribution width—standard deviation (RDW-SD) and Body mass index (BMI).

### Foeto-maternal outcome recorded among study participants

**Fig 2:**
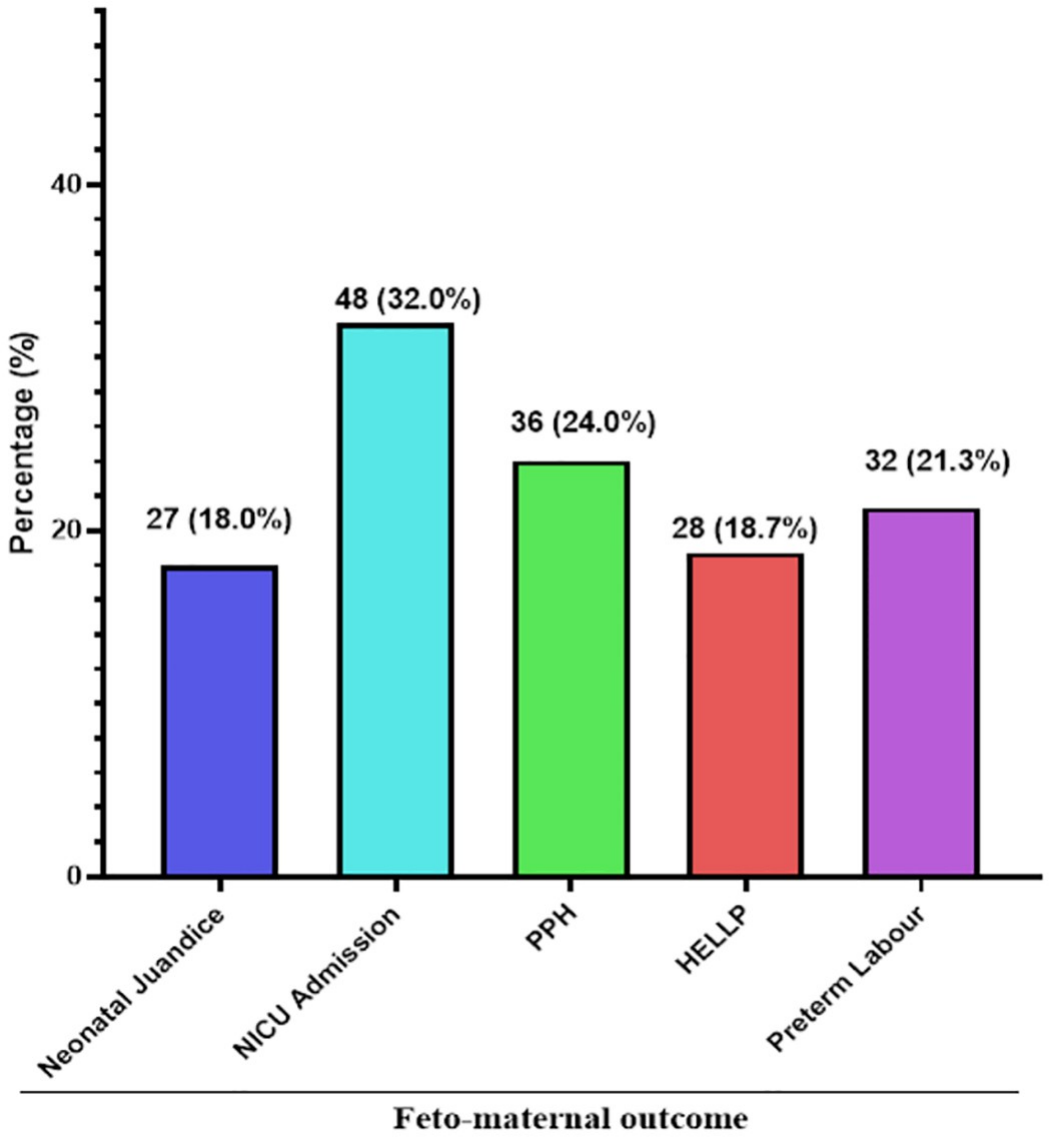
Foetal and maternal outcomes of study participants. PPH: Post-partum haemorrhage, HELLP: Hemolysis, Elevated Liver enzymes and Low Platelets. Some participants had multiple foeto-maternal outcome.

As shown in Fig 2, of the 150 preeclamptic women, more than one-third experienced NICU admission (32.0%) being the most common foeto-maternal outcome. The proportion of participants that experienced PPH, preterm labour, HELLP, and neonatal jaundice were 24.0%, 21.3%, 18.7%, and 18.0% respectively (Fig 2).

### Association between haemoglobin variants and foeto-maternal outcomes

Tables 4 and 5 display the association between Haemoglobin variants and foeto-maternal outcomes among the study participants. After adjusting for maternal age, gravidae and residence in a multivariate logistic regression model, participants with HbAS, HbAC, HbCC, HbSC, HbSS and having at least of copy of S or C were associated with increased odds of neonatal jaundice, NICU admission, PPH and HELLP syndrome compared to participants with HbAA (Tables 4 and 5).

**Table 4:** Association between foetal outcomes and haemoglobin variants.

Phenotype expression	Neonatal jaundice		NICU Admission	
	OR (95% CI)	*p*-value	OR (95% CI)	P-value
**Model 1 (Unadjusted)**				
HbAA	1 (referent)		1 (referent)	
HbAS	13.9 (4.4–43.4)	**<0.0001**	21.1 (6.5–68.0)	**<0.0001**
HbAC	1.3 (0.3–6.9)	0.7270	4.3 (1.3–15.9)	**<0.0001**
At least a copy of S	14.5 (5.0–42.2)	**<0.0001**	17.1 (5.8–50.2)	**<0.0001**
At least a copy of C	1.6 (0.4–6.7)	0.5000	3.4 (1.2–14.7)	**<0.0001**
HbCC, SC, SS	8.1 (2.1–31.5)	**0.001**	22.8 (5.6–92.4)	**<0.0001**
**Model 2 (Adjusted)**				
HbAA	1 (referent)		1 (referent)	
HbAS	14.5 (4.5–47.0)	**<0.0001**	22.7 (6.8–76.6)	**<0.0001**
HbAC	1.2 (0.2–6.4)	0.8670	4.8 (1.2–19.7)	**<0.0001**
At least a copy of S	15.4 (5.1–46.9)	**<0.0001**	18.8 (6.1–57.9)	**<0.0001**
At least a copy of C	1.4 (0.3–6.1)	0.6390	5.1 (1.4–19.1)	**<0.0001**
Hb CC, SC, SS	7.2 (1.8–29.3)	**0.006**	26.4 (5.9–117.8)	**<0.0001**

Crude and adjusted logistic regression analysis models to assess association between foetal outcomes such as NICU admission and Neonatal jaundice and Haemoglobin variants such as HbAA, HbAS, HbAC, at least a copy of HbS and at least a copy of HbC. Adjusted for Age, number of births and residence.

**Table 5:** Association between haemoglobin variants and foeto-maternal outcomes among the study participants.

Phenotype expression	PPH		HELLP Syndrome		Pre-term birth	
	OR (95% CI)	*p*-value	OR (95% CI)	*p*-value	OR (95% CI)	*p*-value
**Unadjusted model**						
HbAA	1 (referent)		1 (referent)		1 (referent)	
HbAS	23.0 (6.5–80.9)	**<0.0001**	28.2 (7.9–100.3)	**<0.0001**	28.7 (8.4–99.0)	**<0.0001**
HbAC	25.9 (7.2–93.0)	**<0.0001**	3.5 (0.8–16.2)	0.106	5.5 (1.5–20.6)	**0.0110**
At least a copy of S	28.2 (8.5–94.0)	**<0.0001**	33.4 (9.9–112.6)	**<0.0001**	39.9 (12.0–132.5)	**<0.0001**
At least a copy of C	18.8 (5.6–62.7)	**<0.0001**	3.8 (0.9–15.3)	0.0640	4.1 (1.1–14.7)	**0.0320**
Hb CC, SC, SS	26.3 (6.1–113.1)	**<0.0001**	26.3 (6.1–113.1)	**<0.0001**	21.7 (5.3–89.2)	**<0.001**
**Adjusted for covariates**						
HbAA	1 (referent)		1 (referent)		1 (referent)	
HbAS	24.6 (6.7–90.4)	**<0.0001**	29.2 (8.0–106.5)	**<0.0001**	29.8 (8.5–104.5)	**<0.0001**
HbAC	30.8 (8.0–117.7)	**<0.0001**	3.6 (0.8–16.9)	0.106	5.8 (1.4–21.7)	**0.0150**
At least a copy of S	31.2 (9.0–108.4)	**<0.0001**	35.4 (10.2–122.7)	**<0.0001**	40.6 (12.0–136.8)	**<0.0001**
At least a copy of C	21.8 (6.2–77.5)	**<0.0001**	3.6 (0.9–15.0)	0.0810	4.2 (1.1–15.9)	**0.0370**
Hb CC, SC, SS	33.6 (7.0–160.6)	**<0.0001**	28.7 (6.2–132.0)	**<0.0001**	24.8 (5.6–110.3)	**<0.001**

Crude and adjusted logistic regression analysis models to assess association between maternal outcomes such as PPH, HELLP Syndrome and Pre-term birth and Haemoglobin variants such as HbAA, HbAS, HbAC, at least a copy of HbS and at least a copy of HbC. PPH: Post-partum haemorrhage, HELLP: Hemolysis, Elevated Liver enzymes and Low Platelets

## Discussion

Haemoglobin variants such as HbAS and HbSS are common in Africans and OS has been well described among pregnant women with preeclampsia [20, 21]. However, it is unclear whether haemoglobin variants coupled with OS modifies risk of adverse foeto-maternal outcome in pregnant women with preeclampsia. In this study, we observed that haemoglobin variants contributed to adverse foeto-maternal outcomes preeclamptic women. Participants with HbAS, HbAC, having at least of copy of S or C and those with HbCC, SC, SS were associated with increased odds of NICU admission, PPH and HELLP syndrome. However, except for vitamin C, oxidative stress markers were generally similar across the haemoglobin variants.

Haemoglobin variants are common in western and central Africa where there is about 25% sickle cell trait and 1–3% sickle cell disease [22, 23]. In this study, the distribution of haemoglobin AA, AS, AC, CC, SS and SC phenotypes were 66.0%, 13.3%, 12.7%, 3.3%, 3.3% and 1.3%, respectively. Our study findings is not different from a study by Umoh *et al*. [24], who reported 78.7% of HbAA and 19.6% of HbAS among Nigerians. With respect to the HbS, it was noted in our study that the proportions of HbAS, HbSC, and HbSS were 13.3%, 1.3% and 3.3% respectively. Umoh et al. [24], reported 19.6% of HbAS, 0.2% of HbSC and 1.5% of HbSS respectively among Nigerians. The proportion of homozygous HbSS (3.3%) was higher than the heterozygous HbSC (1.3%) participants. Participants with heterozygous state tend to have mild complications of anaemia than their homozygous HbSS counterparts. This observation is worrying especially when majority of the study participants reside in rural communities where access to healthcare and poor road network presents further challenges to healthcare delivery; coupled with the fact that preeclampsia needs immediate attention to avoid complications. This study found haemoglobin C variant as the second most common variant after the haemoglobin S. This finding confirms the findings by Piel *et al*. [25], who reported high HbC and its distribution among newborns in Africans. A possible explanation for this could be due to the proximity of Bono and Bono East regions to the three Northern regions of Ghana where haemoglobin variant C accounts for a significant proportion of their population [26] and thus interethnic marriage and migration from the three regions of the northern Ghana to Brong Ahafo could have accounted for this similarity.

The oxidative stress markers were compared across the different haemoglobin variants. The mean vitamin C levels were higher in patients with at least a copy of HbS variant than those with at least a copy of HbC variant. Ascorbic acid presents one of the most important anti-oxidants, exerting beneficial effects by an inhibition of lipid peroxidation and by reducing endothelial dysfunction [27]. This means the antioxidant capacity of preeclamptic women is higher among those who express at least a copy of HbS such as S and AS, than in those with a copy of HbC such as CC and AC. Since preeclampsia is a systemic disease in pregnancy and often associated with inflammation, it is possible that pregnant women with preeclampsia who express at least a copy of HbS would possibly deal with inflammation better than those with at least a copy of HbC variant in a responsive feedback mechanism and hence higher levels of vitamin C.

Of the foeto-maternal outcomes assessed among preeclamptic women, this study found neonatal jaundice and Neonatal Intensive Care Unit (NICU) (32.0%) admission as the most foetal complications recorded followed by PPH (24.0%), preterm delivery (21.3%), HELLP (18.7%), and neonatal jaundice (18.0%). This study finding is comparable to Kawakita *et al*. [28] and Gauchan *et al*. [29] who reported similar prevalence in USA and Nepal repectively.

With regards to the association between haemoglobin variants and foeto-maternal outcome, the neonates of mothers with HbAS phenotype or those with at least a copy of HbS expression were over 14 and 15 times more likely to have neonatal jaundice respectively. Again, compared with individuals with HbAA, a copy of either HbS or HbC expression among pregnant women with preeclampsia was associated with increased odds of NICU admissions (Table 4). Our findings is similar to studies by Elenga *et al*. [30], in French Guiana in Northern South America and Larrabee and Monga [31], in the United States of America, who found that, pregnant women with the homozygotic haemoglobin variants (HbSS), and some double heterozygous conditions, such as HbSC and HbAS, carry a higher risk of pregnancy-related complications. This is worrying due to the fact that the HbAS was the most common haemoglobin variant found in this study. The defective structure of red cells due to sickle cell disorders, results in increased haemolysis, leading to the release of free haemoglobin and subsequent jaundice [32, 33].

Moreover, compared with mothers with HbAA, a copy of either HbS or HbC expression among pregnant women with preeclampsia was associated with increased odds of NICU admissions (Table 4). This finding is consistent with the findings of Wilson *et al*. [5], who observed higher rates of transfers of infants to NICU at the Korle-Bu Teaching Hospital in Ghana due to fetal complications of HbSS compared with those without HbSS. Though the mechanism is unclear, it may be due to the neonate inheriting a copy of the abnormal HbS gene which predisposes them to increased haemolysis that aggravates the known haemolysis in newborns.

In this study, preeclamptic women who expressed at least a copy of HbS or HbC variant had increased folds of PPH. Our finding contradicts the findings of Boafor *et al*. [34], who reported no significant association between post-partum haemorrhage in women who have sickle cell disease compared with women who do not have sickle cell disease. This difference in association may be due to the low number of cases in their analysis, three (3) as against thirty-six, (36) cases reported in our study. Although the mechanisms for preterm delivery among women with sickle cell disease remains unknown, some studies have shown an increased risk of preterm delivery among women with sickle cell disease [35, 36]. This current study also reported a significant association between the risk of preterm delivery and participants that expressed a copy of the HbS or HbC variant (Table 5).

One unfavorable consequence of preeclampsia is the occurrence of HELLP syndrome. We observed that preeclamptic women who expressed at least one copy of HbS variant had increased folds of having HELLP syndrome compared to women with HbAA. Such patients need to be treated in a well-equipped hospital with facilities such as laboratories capable of conducting laboratory investigations such as complete blood count, liver function tests, renal function tests, serum uric acid tests and complete blood count as part of a comprehensive assessment of patients presenting with such conditions.

The strength of the present study is that biomarkers of oxidative stress and its association with haemoglobin variants and adverse foeto-maternal outcome among preeclamptic women is the first to be conducted in a Ghanaian population.

Despite the revealing nature of this study, the study had few limitations. Firstly, the study could not screen neonates of mothers (study participants) for their haemoglobin variants status to verify if the neonatal jaundice observed in our study was due to inherited genes. Secondly, the oxidative stress state at various stages of pregnancy could not be measured because our study participants were either in their second or third trimester when they presented in an emergency condition. Thirdly, most of study participants reported in an emergency presentation therefore measuring and comparing gestational age and weight of delivered baby to the weight measured during the time of presentation (intrauterine) was not practicable. Lastly, a longitudinal cohort study will be required to establish casual-and-effect relationship and thus a cohort study is recommended for future outlook.

## Conclusion

Reduced levels of vitamin C are common among preeclamptics with at least one copy of the HbC variant. Haemoglobin variants in preeclampsia contribute to adverse foeto-maternal outcome with haemoglobin S variants being the mostly influencing factor for PPH, HELLP, preterm labour, NICU admission and neonatal jaundice. It is therefore incumbent on clinicians to monitor and create targeted preventive measures for preeclamptic women with haemoglobin variants to avoid occurrence of adverse pregnancy outcome. Again, the reduced vitamin C indicates the need for antioxidant supplementation among preeclampsia women with sickle cell condition.
